# OTME-23. Single-cell transcriptomic and epigenomic immune landscape of isocitrate dehydrogenase stratified human gliomas

**DOI:** 10.1093/noajnl/vdab070.074

**Published:** 2021-07-05

**Authors:** Pravesh Gupta, Minghao Dang, Dapeng Hao, Krishna Bojja, Tuan M Tran, Huma Shehwana, Carlos Kamiya-Matsuoka, Jianzhuo Li, Alessandra Audia, Cynthia Kassab, Martina Ott, Joy Gumin, Sanaalarab Alenazy, Alicia Goldman, Sahil A Seth, Atul Maheshwari, Veerakumar Balasubramaniyan, Brian Vaillant, John F de Groot, Frederick F Lang, Antonio Iavarone, Nicholas E Navin, Amy B Heimberger, Linghua Wang, Krishna P Bhat

**Affiliations:** 1 Department of Translational Molecular Pathology, The University of Texas MD Anderson Cancer Center, Houston, TX, USA; 2 Department of Genomic Medicine, The University of Texas MD Anderson Cancer Center, Houston, TX, USA; 3 Department of Genetics, The University of Texas MD Anderson Cancer Center, Houston, TX, USA; 4 Department of Bioinformatics and Computational Biology, The University of Texas MD Anderson Cancer Center, Houston, TX, USA; 5 Department of Neuro-Oncology, The University of Texas MD Anderson Cancer Center, Houston, TX, USA; 6 Department of Neurosurgery, The University of Texas MD Anderson Cancer Center, Houston, TX, USA; 7 Graduate School of Biomedical Sciences, The University of Texas MD Anderson Cancer Center, Houston, TX, USA; 8 Department of Neurology and Neuroscience, Baylor College of Medicine, Houston, TX, USA; 9 Department of Neurology, University of Texas at Austin, TX, USA; 10 Institute for Cancer Genetics, Department of Pathology and Cell Biology, Department of Neurology, Herbert Irving Comprehensive Cancer Center, Columbia University Medical Center, New York, NY

## Abstract

The brain tumor immune microenvironment (TIME) continuously evolves during glioma progression, but a comprehensive characterization of the glioma-centric immune cell repertoire beyond a priori cell states is uncharted. In this study, we performed single-cell RNA-sequencing (scRNA-seq) and single cell- Assay for Transposase-Accessible Chromatin using sequencing (sc-ATAC-seq) on ~100,000 tumor-associated immune cells from seventeen isocitrate dehydrogenase (IDH) mutation classified primary and recurrent human gliomas and non-glioma brains (NGBs). Our analyses revealed sixty-two transcriptionally distinct myeloid and lymphoid cell states within and across glioma subtypes and we noted microglial attrition with increasing disease severity concomitant with invading monocyte-derived cells and lymphocytes. Specifically, certain microglial and monocyte-derived subpopulations were associated with antigen presentation gene modules, akin to cross-presenting dendritic cells (DCs). We identified cytotoxic T cells with poly-functional cytolytic states mostly in recurrent IDH-wt gliomas. Furthermore, ligand-receptor interactome analyses showed a preponderance of antigen presentation and phagocytosis over the checkpoint axis in IDH-wt compared to IDH-mut gliomas.

Additionally, our sc-ATAC-seq analyses revealed differences in regulatory networks in NGBs, IDH-mut and IDH-wt glioma associated immune cells. In particular, we noted abundant usage of inflammatory transcription factors (TFs) as exemplified by Nuclear factor kappa B and Activator Protein-1 TF family in IDH-wt microglia when compared with microglia from IDH-mut and NGBs. Unique features such as amplification of 11- Zinc Finger Protein accessibility were restricted to monocyte derived cells and were not observed in microglia. Finally, sc-ATAC-seq profiles of CD8^+^ exhausted T cells from IDH-wt showed strong enhancer accessibility on Cytotoxic T-lymphocyte-associated protein 4, Layilin and Hepatitis A Virus Cellular Receptor 2 but no enrichment on PDCD1 (gene encoding Programmed cell death protein 1) was seen. In summary, our study provides unprecedented granular detail of transcriptionally defined glioma- specific immune contexture that can be exploited for immunotherapy applications.

This study in K.B. laboratory was supported by the generous philanthropic contributions to The University of Texas (UT) MD Anderson Cancer Center (MDACC) Moon Shots Program™, Marnie Rose Foundation, NIH grants: R21 CA222992 and R01CA225963. This study was partly supported by the UT MDACC start-up research fund to L.W. and CPRIT Single Core grant RP180684 to N. E. N.

